# Supplementary material for: Application of Machine Learning in the Quantitative Analysis of the Surface Characteristics of Highly Abundant Cytoplasmic Proteins: Toward AI-Based Biomimetics
Source: Biomimetics (Basel). 2024 Mar 6;9(3):162. doi: 10.3390/biomimetics9030162 (PMC10967800; doi:10.3390/biomimetics9030162)
Supplement: Supplementary file 1 [file biomimetics-09-00162-s001.zip › biomimetics-2798824-supplementary.pdf]

Supplementary Information

Application of machine learning in the quantitative analysis of surface characteristics of highly abundant cytoplasmic proteins: Toward AI-based biomimetics

Jooa Moon[1], *Guanghao Hu*[1], *and Tomohiro Hayashi*[1,2*]

[1] Department of Materials Science and Engineering, School of Materials and Chemical Technology, Tokyo Institute of Technology, 4259 Nagatsuta-cho, Midori-ku, Yokohama, Kanagawa 226-8502, Japan

[2] The Institute for Solid State Physics, The University of Tokyo, 5-1-5, Kashiwanoha, Kashiwa, Chiba 277-0882, Japan

*corresponding author: tomo@mac.titech.ac.jp

Contents:

1. List of HAC and extracellular proteins S-1

2. Single-shot hyperparameter S-2

3. Statistical summary of descriptors S-3

4. Calculation of the surface net charge S-4

Table S1. List of Uniprot IDs for the HAC and extracellular proteins.

HAC Proteins				Extracellular Proteins			
P51148	P31946	P62917	P27348	Q2WEN9	P31371	Q12805	P50453
P22392	Q99714	P40429	P62491	Q9BTY2	Q6UWY2	P09668	Q6Q788
Q9Y230	P62258	Q9Y3Y2	Q9Y3I0	P20160	P26022	P61812	P02753
P05787	P48643	P10412	Q07666	P04070	Q8IXL6	O95841	O76096
P12004	P49207	P68032	P61604	P43235	Q8N8U9	P01037	Q6ZMJ4
P62081	P63104	Q15717	O60664	Q9Y5C1	P06702	P00748	P23510
P07437	P60866	P62987	P46776	P05230	Q9BXJ7	O95393	P05019
P38159	Q01628	P40925	P68371	Q9UMX5	Q8WWY8	P08962	Q8NDZ4
P63241	Q01518	Q8WY22	P49006	Q8WX77	O95633	Q30201	P06858
P83731	P05141	Q02543	Q9BUF5	P01619	P02748	Q9Y287	P25311
Q99832	P51991	P37108	Q71U36	O15496	Q9H1Z8	P01591	P01135
P62269	Q14847	P84090	Q15102	Q15046	P34096	Q96HE7	P02655
P54652	P35268	Q15365	Q07020	O75718	P08637	O00253	P01588
P25788	P31943	P84077	P62736	P01008	Q9NPH9	P29459	P13385
Q96AE4	P49721	P63261	P35998	P00738	M5A8F1	P20800	Q6UXH8
P47756	P15311	P42766	P62854	P01584	P28799	P05154	P54317
P14866	P40227	P25398	P08621	P01011	P18065	Q12794	Q29983
Q93077	P62910	P78371	P13693	Q9BZM5	Q00604	P24387	P20062
P07737	Q99436	P62318	O00299	P35030	Q6P1S2	Q08629	P01036
Q13162	Q15005	P12277	P54577	P20142	O15123	P02790	P03952
O15347	Q9Y5S9	P35754	Q02878	Q9H1E1	O95389	O76076	Q9UBV4
P62913	Q15942	P52565	Q9Y3C6	P14138	Q13253	P08253	P01019
Q00765	P61088	P06454	P06753	P69905	Q96LR4	Q9GZV9	P13521
P05114	P62899	Q9NPJ3	P13489	P17936	P00797	Q9BY76	P04141
Q05639	P62805	P61981	P36542	P03950	P02647	P11150	Q08380
P55084	P62701	P31948	P04792	P00734	Q15389	P61626	Q6P4A8
P61254	P32119	Q04941	P39023	Q96FQ6	P41221	Q15848	O00584
Q14103	P62979	P18124	Q92522	P12724	P08246	P01033	Q6UX06
P46777	P30040	P07195	P30041	P09228	P04004	Q969H8	P21246
P23528	P39687	P82979	Q15185	Q99988	P01042	P05305	Q6EMK4
Q00325	P68133	Q15233	Q9Y2T7	P02649	P15309	Q99574	P01375
Q01629	P51571	Q14247	Q13148	P56704	Q14116	P00740	
P84098	P29966	Q9H444	O00264	P00749	Q6FHJ7	P18428	
P0CG47	Q9BV40	Q16658	P84103	Q9BWP8	P55000	Q5SY68	
P17987	P50991	Q16543	P08670	P41222	Q14393	Q9UBU3	
P62829	Q9NX24	P06733	P62241	P11597	Q8N474	Q96DR5	
P55769	Q07955	Q13509	P26373	P27169	Q96LT7	P16519	

P62826	P16402	O14979	Q6EBC2	Q9BZP6	P29460
Q9NX63	P62424	Q15366	P04746	Q96HF1	P01876
P31942	Q15056	Q9H3N1	P20933	K9M1U5	P09172
P38646	P28074	P49773	Q86UU9	P02750	P36222
P61978	P09936	Q6FI13	P05231	P16233	P05090
Q15293	Q07065	P60709	Q9NRS4	P01859	P04054
P52597	Q969M3	O95168	Q13790	P07225	P02763
Q9ULV4	Q92597	P20671	Q16568	Q12846	Q9GZN4
P61204	P11021	P62266	Q9BWS9	P04085	P09543
P00558	Q6PEY2	P09211	P03951	P01857	P19876
P84243	P60900	P30086	P02675	P05121	P08697
P04844	P17096	P68363	Q8TEU8	Q9BX67	P27352
P26038	P15531	Q96GD0	Q9NZK5	P25774	P02749
P06748	Q96QV6	P80723	P14174	O75629	P18075
Q99497	P13164	P62879	P22894	P01308	P30046
P46778	P04632	P0DME0	Q6UW32	P02743	P0DTE8
P02545	Q9NS69	P0DP23	Q9Y646	Q15465	Q8N423
P50454	P52926	P35908	O14791	Q9H2A7	Q6P5S2
P55809	P10620	P30085	P23280	Q12907	P10909
P52209	O15143	Q9UJU6	P29466	Q9H3R2	P68871
Q9Y3U8	Q01105	Q13151	Q8NBP7	Q9P0G3	Q9Y6Z7
Q8NC51	P00505	P46783	P43652	Q9BT56	P05413
Q99426	Q15084	P16989	O96009	P01258	Q9UBU2
P05783	P61353	Q9BVA1	Q99075	P17405	P01225
Q02539	P18085	P23193	P12273	P01877	P02768
P68431	P30043	P61586	Q7Z5A9	Q9NRA1	P22466
P08865	P16403	Q9Y281	P16581	P05452	P0DUB6
Q99729	Q7RTV0	P61313	P07711	Q8NCC3	O60687
P62888	P40926	P31949	Q99969	P18510	P13726
P07910	P0DPH7	P50502	P03971	P02760	P19438
P47914	P46779	Q16695	P09038	P35318	Q13287
P16104	P04075	Q13409	O95925	P01009	P11226
P27824	P22061	P62750	Q13231	P40199	P15085
P52907	P10809	P20700	Q9BXJ3	Q96EG1	Q6UY27
P04406	P00441	P61158	Q5W188	P28300	P0DTE7
Q13885	Q13247	Q9NZZ3	Q6X4U4	Q15582	P01137
Q04837	P50914	O75083	P06727	P02679	P17813
P04843	O43684	P06576	Q9NRJ3	A5D8T8	Q7Z5A8
P63244	Q562R1	P61247	Q7Z5A7	Q9UBR2	P01034

P26368	P38117	P09497	P04180	Q6UWQ7	P14555
P05388	P50395	Q71DI3	Q9BXJ1	Q9H9S5	Q7Z5L7
P09622	Q01130	Q16629	Q9Y2Y6	O14793	Q1L6U9
P08729	P17844	P23526	P08236	P07602	O94907
P40121	O75396	Q9BRP8	P07498	O00300	P20851
Q06830	P09651	P49411	Q6ZVN8	P19652	Q96MK3
Q13185	P63267	P18621	P12644	Q9H3U7	P31025
O14828	P13667	P13804	P81605	Q9Y3A2	P22301
P04350	P53999	P12956	O95388	Q6UXB1	Q6UX52
Q01844	Q9UKY7	Q9Y266	P04003	P56705	P07288
Q03252	Q9UKM9	P05198	Q13510	Q96CG8	P02741
P14314	P39019	P30101	P07858	P05362	P31997
P36578	P68104	P62316	P01583	Q8TB73	P41271
P04264	P15559	P09496	Q92484	P10646	P20366
P00352	P84095	P0C0S8	Q9Y264	O15520	Q5TCH4
P27695	P11142	Q9BQE3	O95994	P13473	Q9NP55
P35080	P60174	P48735	Q92820	Q96QR1	P01215
P16401	P18077	P26599	P04083	P01241	P05089
Q9UBQ0	P46781	P00338	P06850	Q93091	P03973
P50990	P16949	P14209	Q8TAD2	O15537	O14944
Q99623	P25786	P23396	O75356	Q96IU2	Q9Y5X9
Q15907	Q56VL3	Q8IUE6	P05814	P0DOX3	P40313
P29373	Q9P258	P21796	Q96S42	P12643	P08311
P67936	P28072	P49368	P48740	P01871	Q99972

Table S2. Hyperparameters of machine learning models in a single-shot trial.

Model	Full Name	Hyperparameters
KNN	k-Nearest Neighbors	{'algorithm': 'auto', 'metric': 'manhattan', 'n_neighbors': 11, 'weights': 'distance'}
LR	Logistic Regression	{'C': 0.1, 'penalty': 'l2', 'solver': 'liblinear'}
RF	Random Forest	{'max_depth': 10, 'max_features': 'sqrt', 'min_samples_split': 2, 'n_estimators': 1000}
SVM	Supported Vector Machine	{'C': 1.0, 'gamma': 'scale', 'kernel': 'rbf'}

Table S3. Statistical summary of surface descriptors for all protein data set.

	s_phobic _avg	s_neg_area_ avg	s_pos_area_ avg	norm_s_b	FD	s_bs	s_do
Extracellular Proteins							
count	331						
mean	-1.101	0.137	0.193	0.231	2.192	13.009	52.988
std	0.366	0.043	0.048	0.102	0.060	9.479	12.690
min	-1.998	0.024	0.061	-0.049	2.066	0	7.23
25%	-1.3365	0.1135	0.1635	0.159	2.144	5.694	45.2715
50%	-1.148	0.137	0.19	0.251	2.190	11.609	54.704
75%	-0.899	0.1605	0.221	0.298	2.235	19.968	60.874
max	0.719	0.305	0.364	0.491	2.372	47.337	88.165
Highly Abundant Cytoplasmic (HAC) Proteins							
count	337						
mean	-1.569	0.170	0.257	0.185	2.180	8.691	50.151
std	0.452	0.076	0.090	0.098	0.069	8.643	16.337
min	-2.74	0.022	0.031	-0.02	2.045	0	1
25%	-1.854	0.112	0.195	0.126	2.117	2.578	39.872
50%	-1.6	0.169	0.236	0.192	2.178	6.733	48.05
75%	-1.385	0.22	0.309	0.26	2.232	12.685	58.326
max	0.294	0.555	0.495	0.512	2.325	57.663	99.187

*Full name of each descriptor:

s_phobic_avg: Average Surface Hydrophobicity

s_neg_area_avg: Average Negatively Charged Surface Area

a_pos_area_avg: Average Positively Charged Surface Area

norm_s_b: Average Normalized Surface b-factor

FD: Average Surface Roughness

s_bs: Surface Beta Strands Composition

s_do: Surface Disordered Regions Composition

Table S4. pKa Values used to calculate net charge [51].

1-letter code	Amino Acid	pK_a Values		
		pK_{a1}(-COOH)	pK_{a2}($-NH_3^+$)	pK_{aR}(R group)
A	Alanine	2.35	9.87	
C	Cysteine	2.05	10.25	8.00
D	Aspartic Acid	2.10	9.82	3.86
E	Glutamic Acid	2.10	9.47	4.07
F	Phenylalanine	2.58	9.24	
G	Glycine	2.35	9.78	
H	Histidine	1.77	9.18	6.10
I	Isoleucine	2.32	9.76	
K	Lysine	2.18	8.95	10.53

L	Leucine	2.33	9.74	
M	Methionine	2.28	9.21	
N	Asparagine	2.02	8.80	
P	Proline	2.00	10.60	
Q	Glutamine	2.17	9.13	
R	Arginine	2.01	9.04	12.48
S	Serine	2.21	9.15	
T	Threonine	2.09	9.10	
V	Valine	2.29	9.72	
W	Tryptophan	2.38	9.39	
Y	Tyrosine	2.20	9.11	10.07

Surface net charge was calculated using Table S3 under physiological conditions (pH=7). General expressions derived from Henderson-Hasselbalch equation calculated negative (Q^-) and positive (Q^+) charge as follows [52]:

$$Q^- = \frac{-1}{1 + 10^{-(pH-pKa)}}$$

$$Q^+ = \frac{1}{1 + 10^{+(pH-pKa)}}$$

Thus, surface net charge of protein was calculated for all the surface residues with the following general expression:

$$Q_{protein} = \sum Q^- + \sum Q^+$$

Where Q^- includes C-terminus and negatively charged R groups, and Q^+ includes N-terminus and positively charged R groups.
